# A Systematic Review on Current Trends in the Treatment of Chronic Hepatitis B to Predict Disease Remission and Relapse

**DOI:** 10.7759/cureus.32247

**Published:** 2022-12-06

**Authors:** Samia Rauf R Butt, Travis Satnarine, Pranuthi Ratna, Aditi Sarker, Adarsh Srinivas Ramesh, Carlos Munoz, Dawood Jamil, Hadrian Hoang-Vu Tran, Mafaz Mansoor, Safeera Khan

**Affiliations:** 1 General Practice, California Institute of Behavioral Neurosciences & Psychology, Fairfield, USA; 2 Pediatrics, California Institute of Behavioral Neurosciences & Psychology, Fairfield, USA; 3 Internal Medicine, California Institute of Behavioral Neurosciences & Psychology, Fairfield, USA

**Keywords:** peg-interferon, nucleotide analogs, late-relapse, treatment free remission, remission, chronic hepatitis b (chb)

## Abstract

Despite decreasing the prevalence of chronic hepatitis B (CHB), it is still a major health care challenge. Current antiviral regimens aim to suppress hepatitis B virus (HBV) deoxyribonucleic acid (DNA) activity to prevent the risk of hepatic decompensation, liver cirrhosis, and hepatocellular carcinoma (HCC). Currently, pegylated interferon (Peg-IFN) and nucleos(t)ide analogs (NA) are the first-line choices of drugs. Peg-IFN is now discontinued due to its mode of application and side effects. NA is used once daily to suppress HBV DNA activity but has little effect on covalently closed circular DNA (cccDNA), so continuous long-term therapy is required to suppress HBV DNA. Due to this effect, disease remission, relapse, and even clinical flare are common phenomena after the end of treatment (EOT). This review aimed to analyze the current regimens for treating chronic hepatitis B. Their mode of action, duration of treatment, and events after stopping therapy. The review was performed using the preferred reporting items for systematic reviews and meta-analysis (PRISMA) 2020 guidelines. A search was undertaken in PubMed, PubMed Central, Google Scholar, and ScienceDirect. Screening of articles was carried out to find relevant and appropriate articles. Articles were then quality-checked before inclusion.

Our analysis showed that long-term finite therapy with nucleoside analogs could improve clinical outcomes and suppress viral DNA activity. However, a functional cure, loss of hepatitis B surface antigen (HBsAg), is rarely achieved. The decision to end treatment depends on quantitative HBsAg level (qHBsAg), alanine aminotransferase (ALT), HBV DNA (deoxyribonucleic acid), hepatitis B e antigen (HBeAg), and fibrosis assessment. It is concluded that patients with HBeAg negative without cirrhosis can be easily withdrawn from treatment if they have long-term viral remission and a high HBsAg loss rate. However, patients with positive HBeAg should continue treatment because there is a high chance of disease relapse and even acute flare. To predict whether patients will benefit from EOT, some immunomodulatory markers are studied, including interleukin (IL-20, IL-8), fas ligand (FASGL), and IFN gamma. Although these factors are reliable, none pose an independent effect on disease remission. Combination therapy (IFN alpha + oral nucleoside analogs) is promising but has clinical shortcomings.

## Introduction and background

Hepatitis B virus (HBV) is one of the most serious and prevalent diseases, even with the introduction of a vaccine in 1982. There are still 290 million people worldwide, with the majority residing in the Asia-Pacific region [1]. In countries where HBV is endemic, there are higher chances of developing chronic hepatitis. Among these, perinatal transmission is the most common cause of infection worldwide, even in the United States of America (USA). This is especially true for those who have not received the HBV vaccine at birth, were born to HBV-positive mothers who didn't receive appropriate immunoprophylaxis, or are immigrants [2].

Chronic hepatitis B (CHB) is marked by persistent HBsAg (surface antigen of the hepatitis B virus) for six months or more. It's a global concern as it can lead to complications of liver cirrhosis, hepatocellular carcinoma, and hepatic decompensation if left untreated [3]. Loss of HBsAg is considered a functional cure and complete remission, which is rarely achieved with the current regimen. Active viral genome replication is the hallmark of disease progression and is marked by HBeAg (e antigen of the hepatitis B virus). So, patients who have HBeAg-positive and negative chronic hepatitis B are candidates for anti-HBV therapy [4].

Currently, two regimens are approved for CHB. The first line is pegylated interferon (Peg- IFN-γ), a subcutaneous injection; the other is oral nucleos(t)ide analogs (NA) entecavir (ETV) and tenofovir disoproxil fumarate (TDF). Peg-IFN acts as an antiproliferative and immunomodulatory agent. Whereas NA inhibits viral replication, thus preventing liver necroinflammation and disease progression. However, both the agents do not affect hepatitis B virus DNA (HBV DNA), especially cccDNA, i.e., covalently closed circular DNA in the nucleus. So, treatment cessation almost always leads to viral remission. Thus, an indefinite continuation of NA is necessary for sustained virological response. Treatment duration relies on the desired treatment goals, but there is conflicting data in considering NA cessation [5].

IFN was the only treatment available before the introduction of lamivudine (LAM), but its functional cure after 48 weeks of treatment was less than 5% [6]. It's interesting to see that among HBeAg-positive patients, peg-IFN alone leads to higher (23%) HBeAg seroconversion, but HBsAg loss is (5%) after six months of discontinuation. Considering their variable effect on genotypes, side effects, and subcutaneous administration, they are no longer a favorable treatment drug [7]. However, studies show that PEG-IFN added to a NA can give better results in the long term [8].

Long-term NA therapy may lead to HBsAg loss, but significant seroconversion is seen after stopping NA [9]. A controlled study done on non-cirrhotic HBeAg-negative patients who had received TDF for more than four years showed that upon stopping, only 19% of patients had HBsAg loss, but after three years, 43% achieved viral remission [10].

However, there is a propensity of data to suggest the baseline of stopping NA therapy. The Asian Pacific Association for the Study of Liver Disease (APASL) suggests that among HBeAg-negative patients whose HBV DNA remains undetectable on three separate occasions, each more than six months apart and their ALT is monitored every month in the first three months and then every three months, and have acquired NA treatment for at least two years might stop it [4]. American Association for the Study of Liver Disease (AASLD) suggests indefinite antiviral treatment for HBeAg-negative patients considering the deadly outcome of the disease, financial burden, patient adherence, close monitoring, and provider preference [11].

In light of the evidence suggested by previous studies regarding the use of peg-IFN and nucleotide analogs in treating CHB, physicians are still interested in investigating the endpoint to stop therapy. Monotherapy or combination therapy still has chances of remission, relapse, or flare-up. With relapse, there are chances of deterioration to hepatic decompensation, so close monitoring is crucial. To clarify, we conducted a systematic review to improvise a standard of care and the potential benefit of these therapies in preventing clinical relapse of CHB.

## Review

To stop the progression of chronic hepatitis B to anticipated liver damage, peg -IFN therapy and long-term administration of third-generation nucleotide analogs have been suggested [12]. It's clear that long-term NA therapy will lead to virological remission and histological improvement of the liver. Still, the cost of stopping NA is a high chance of virological relapse [13]. In this systematic review, we aim to analyze multiple studies to clarify antiviral therapy against CHB and its effect on disease remission and relapse, considering the mechanism of action, duration, and host factors.

Method

Methodology and Search Strategy

Our method and results for systematic review are reported according to the preferred reporting items for systematic reviews and meta-analysis (PRISMA) 2020 guidelines following our screening selection [14].

We used PubMed, ScienceDirect, and Google Scholar to look for articles using Medical Subject Headings (MeSH) and keywords to highlight the most relevant reviews and studies for analysis. The keywords included: "Chronic Hepatitis B", "peg-IFN alpha", "oral nucleotide analogs", "remission" and "relapse". We used Booleans to put together the keywords for an algorithm to use in PubMed. The articles were screened to highlight those most relevant to the search question and selected according to the inclusion/exclusion criteria.

Inclusion and Exclusion Criteria

The selection choice was from randomized control trials (RCTs) published from 2018 to 2022. All selected articles were peer-reviewed and published in the English language. Grey literature was excluded. Our selection for eligibility followed the population, intervention, comparison, and outcomes (PICO) model. The inclusion and exclusion criteria are shown in Table 1.

**Table 1:** Showing inclusion and exclusion criteria of the study CHB: Chronic Hepatitis B

Inclusion Criteria	Exclusion Criteria
Adult patients with chronic hep B (age 18 or above). Patients receiving treatment for chronic hep B. The research published in the past five years. Patients taking alpha interferon or nucleotide analogs or combination. Patients from any geographical region of the world. Systematic reviews and observational studies were included.	Children (age less than 18). Pregnant patients with CHB. Transplant patients with CHB. Chronic hep B patients with other co-morbidities (e.g., kidney disease, bone disease) and receiving treatment regimens for other co-morbidities. Patients who developed multiorgan failure from CHB patients with liver cirrhosis and hepatocellular carcinoma who are also receiving treatment for CHB. Studies that were conducted before 2017. Patients with an acute flare-up of hepatitis B. Articles on guidelines.

Data Extraction

The data retrieval and review were completed by two separate researchers independently. In the case of disagreements, the researchers would discuss the data for its relevance and design to eligibility criteria to reach an accord. A third researcher was counseled for objectivity if a decision could not be made.

Critical Appraisal of Studies

We critically appraised our screened articles using the Cochrane risk of bias tool [15]. The bias risk assessment looked at seven causes of potential bias, and a summary was given for each clinical trial in this review in Table 2.

**Table 2:** Quality assessment of randomized control trials using Cochrane bias tool

Article	Random sequence generation –Selection Bias	Allocation of concealment – Selection bias	Blinding of both participants and evaluators – Performance Bias	Blinding of assessment during outcome collection – Detection bias	Incomplete outcome data – Attrition Bias	Selective reporting – Reporting Bias	Other bias/ Comments
Liem et al. 2019[16]	Low risk	Low risk	High risk	Low risk	Low risk	Low risk	Due to the intervention, patients, clinicians, and the study team could not be blinded to treatment allocation.
Suárez et al. 2018[17]	Low risk	Low risk	High risk	Low risk	Low risk	Low risk	
Huang et al. 2021 [18]	Low risk	Low risk	High risk	Low risk	Low risk	Low risk	
Ahn et al. 2021[19]	Low risk	Low risk	High risk	Low risk	Low risk	Low risk	
Hsu et al. 2018[20]	Low risk	Low risk	High risk	Low risk	Low risk	Low risk	
Jeng et al. 2019 [21]	Low risk	Low risk	High risk	Low risk	Low risk	Low risk	
Kranidioti et al.2019[22]	Low risk	Low risk	High risk	Low risk	Low risk	Low risk	
Li et al. 2021[23]	Low risk	Low risk	High risk	Low risk	Low risk	Low risk	
Manolakopoulos et al. 2020[24]	Low risk	Low risk	High risk	Low risk	Low risk	Low risk	

Results

Literature Search and Study Selection

**Figure 1:**
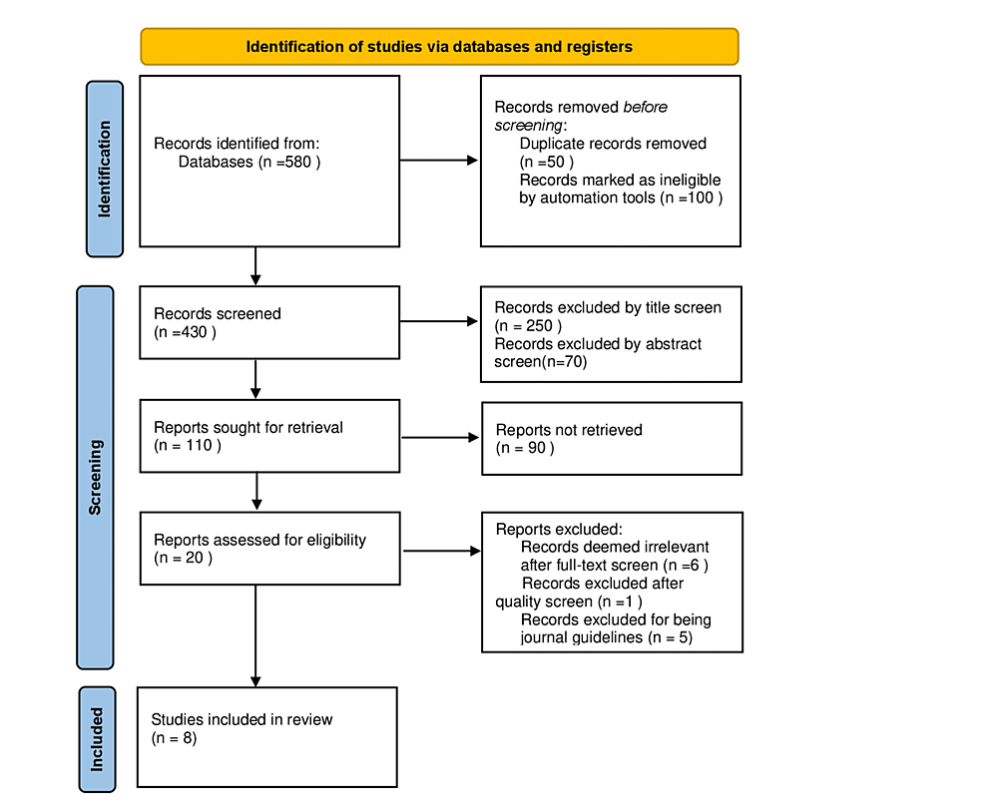
PRISMA 2020 flow diagram for new systematic reviews, which included searches of databases and registers only PRISMA: Preferred reporting items for systematic reviews and meta-analysis

The MeSH strategy generated five hundred eighty articles from keywords, eligibility criteria, and databases. This systematic review was conducted following the preferred reporting items for systematic reviews and meta-analyses (PRISMA) guidelines. Fifty articles were excluded due to duplication. Upon reading the titles and abstracts, 320 articles were excluded. After filtering to include articles from the last five years only, as the guidelines vary consistently, we were left with 20 articles. Of the remaining articles, five of them were journal guidelines, and one didn't meet the quality check criteria. Eight final articles met the criteria and were included. Our PRISMA flow diagram is shown below in Figure 1.

Baseline characteristics: These characteristics and definitions are generalized to all reviewed articles [16-22].

Treatment Indications

If HBV DNA>2000 IU/ml and ALT > two times the Upper Limit of Normal (ULN) or HBV DNA >2000 IU/ml and ALT >ULN with moderate necroinflammatory activity and fibrosis.

Patients were excluded if they had detectable HBV-DNA or positive HBeAg at treatment cessation, presence of liver cirrhosis, co-infection with hepatitis C or human immunodeficiency virus, chronic kidney disease, hepatic decompensation, hepatic, or non-hepatic malignancy, received an organ transplant, or used immunosuppressive drugs in the last few months.

Virological remission: Virological remission is defined as undetectable HBV DNA and negative HBeAg.

Clinical relapse: Clinical relapse was defined as serum ALT greater than two folds the upper limits of normal (40 U/L) plus serum HBV DNA rising above 2000 IU/mL

Virological relapse: Elevation of HBV DNA >2000 IU/ml

Expected outcome: Loss of HBsAg in serum, which was defined as a measurement below the lower limit of detection (0.05 IU/mL)

**Figure 2:**
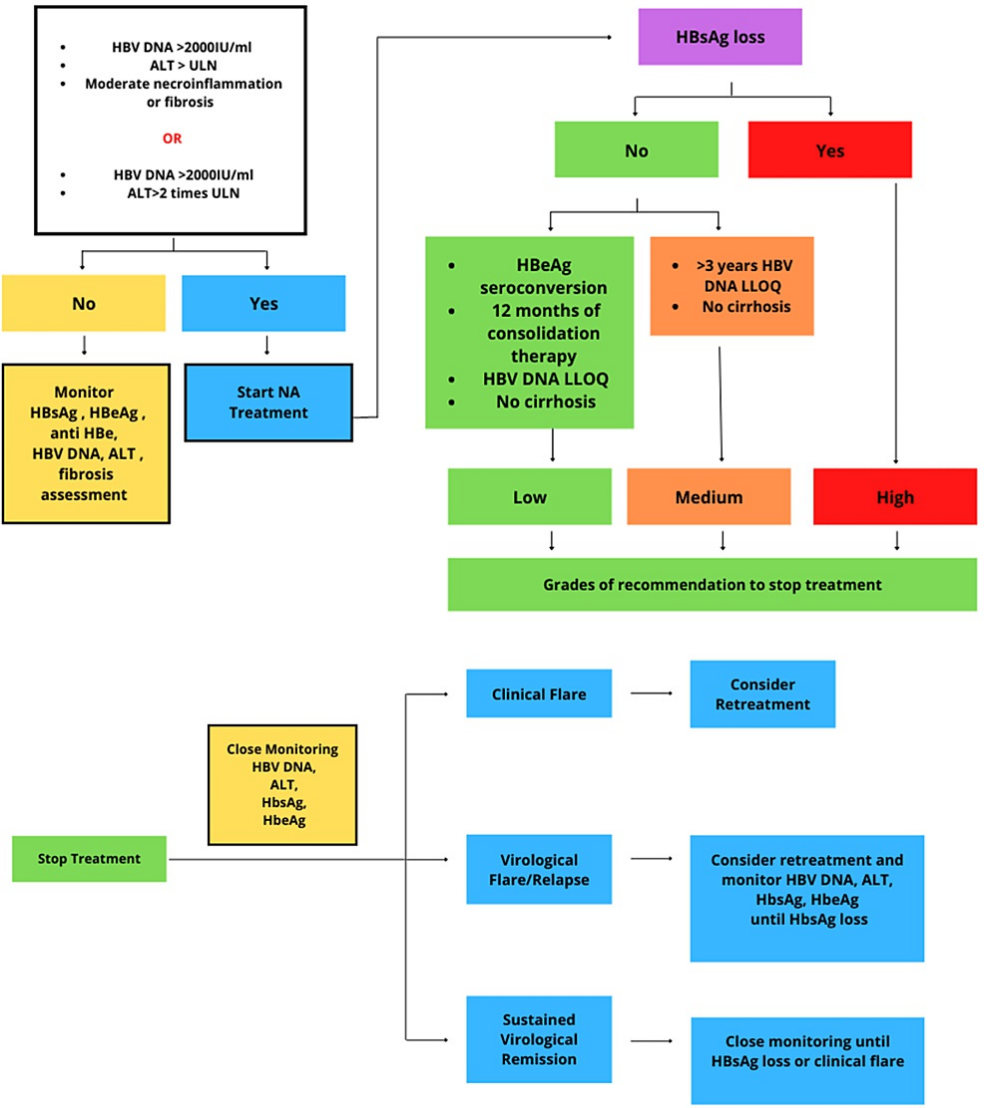
Schematic overview of starting treatment, discontinuing treatment, and typical course observed after treatment discontinuation HBV DNA: Hepatitis B virus deoxyribonucleic acid, ALT: Alanine aminotransferase, ULN: Upper limit of normal, HBsAg: Hepatitis B surface antigen, HBeAg: Hepatitis B e antigen, NA: Nucleoside analog, LLOQ: Low limit of quantity

A systematic review of the management paradigm is shown in Figure 2.

A brief overview of the studies included is given in Table 3.

**Table 3:** Showing baseline characteristics of the studies included. CHB: Chronic hepatitis B, NA: Nucleotide analog, HBsAg: Hepatitis B surface antigen, HCC: Hepatocellular carcinoma, IL-21: Interleukin 21, TDF: Tenofovir disoproxil fumarate, PEG-IFN: Pegylated interferon , FASGL: Fas ligand, CCL4: Chemokine ligands 4

Article	Study design	Inclusion criteria	Intervention	Primary outcome	Secondary outcome	Conclusion
Liem et al. 2019[16]	Randomized control trial	CHB patients, 18 or above, received NA therapy	Patients were randomized in 2:1 to discontinue or continue tenofovir and followed for 72 weeks	27% patients had sustained response, 71% relapsed and 2% achieved HBsAg loss at 72 weeks after stopping NA therapy	_	Stopping NA therapy confers little to no benefit in this study done on the Asian population
Suárez et al. 2018[17]	Prospective cohort	Sixty-nine patients who discontinued treatment with NA after HBs Ag loss	_	In the three-year follow-up, only one patient developed HCC, and one patient in the white population developed reactivation due to immunosuppressive therapy	_	HbsAg clearance is persistent after three years of treatment with NA
Huang et al. 2021 [18]	Prospective cohort	Serum levels of IL-21 were checked in patients at zero, 12, 24, 52, and 104 weeks after stopping entecavir	_	IL-21 has a positive correlation with HBsAg levels. When entecavir was stopped disease relapsed in HBsAg + patients	_	IL-21 acts as an immunomodulator in maintaining disease relapse in HBsAg-positive patients
Ahn et al. 2021[19]	Randomize control trial	_	Four random groups 1) TDF+PEG-IFN for 48 weeks 2) TDF+PEG-IFN for 16 weeks followed by 32 weeks of TDF alone 3) TDF alone for 30 months 4-PEG-IFN alone for 48 weeks	Mean HBsAg loss at 30 months was greater with group A than with any other group	_	Combination therapy of PEG-IFN and NA is better than monotherapy
Hsu et al. 2018[20]	Prospective cohort	One hundred thirty-five CHB patients who achieved viral remission after stopping NA therapy, with negative HBeAg and undetectable HBV DNA	After cessation of NA (entecavir or tenofovir), patients were followed up every three months for 25.9 months. Used SCALE-B scoring system to predict relapse	Sixty-six developed clinical relapse, and eight developed HBsAg loss	Loss of HBsAg occurred in 8.8% of patients within three years	HBsAg and HBeAg are independent factors to predict virological relapse after cessation of NA
Jeng et al. 2019[21]	Prospective cohort	Forty-eight patients who had HBV DNA undetectable and stopped NA therapy two years ago were enrolled. CASE: 36 with viral remission and 12 with viral relapse (not retreated) CONTROL: remaining 12 with HBsAg positive and non-retreated	All patients had HBsAg loss in a five-year follow-up	HBsAg kinetics from the end of therapy to complete loss of HBsAg in HBeAg negative CHB patients, a gradual decline is seen in the first six to seven months and then a 'precipitous decline' in two-three years before the complete loss.	_	_
Kranidioti et al.2019[22]	Randomized control trial	CASE: 33 patients who had stopped NA therapy CONTROL 1: nine patients with HBsAg positive but HBeAg negative (inactive carriers) Control 2: 38 HBeAg negative CHB patients undergoing NA therapy	Peripheral blood mononuclear cell samples for gene expression analysis were collected at the end of therapy and six months after NA discontinuation	Remission was achieved in 47%of patients after discontinuing treatment	Ten out of 21 genes were down-regulated. Out of them, four are IFN-gamma and IL-8. FASLG and CCL4. That shows successful seroclearance and predicts off-treatment remission	_
Li et al. 2021[23]	Randomize control trial	Patients were aged 18-65. HBsAg positive for at least six months and HBeAg positive	Group A: 62 patients- TDF alone for 96 weeks. Group B: 27 patients-TDF alone for 48 weeks, followed by peg-IFN for 48 weeks. Group C: 44 patients-TDF +peg-IFN for 48 weeks	HBeAg seroconversion at week 96 was achieved by 22.5% in group A, 14.82% in group B, and 34.09% in group C	The difference was not statistically significant, p=0.157 at 96 weeks, p=0.72 at week 48 p=0.117 achieved HBeAg clearance	De novo combination therapy is better. Serum level of IP-10 was higher in patients who had HBsAg seroconversion or HBsAg loss
Manolakopoulos et al. 2020[24]	Prospective cohort	HBeAg negative at the start of NA and upon discontinuation. Continuous NA therapy for four years, with the last two years only taking entecavir or tenofovir. HBV DNA was undetectable for at least three years. Absence of advanced liver disease	_	Virological relapse was high, up to 94% at 48 months of stopping NA.	_	Finite therapy of NA leads to disease relapse within three years, and retreatment is needed. However, none of the patients developed liver cirrhosis or decompensation

Discussion

Treatment Goals After Stopping NA Therapy

Since available regimens can only suppress the viral replication but cannot target the cccDNA in the nucleus, relapse is most likely seen after stopping NA in HBeAg-negative patients. The relapse rate varies greatly among studies, and whether all patients need retreatment is also unclear. In one of the studies [17], participants were randomized to stop or continue NA treatment. Seventy-two weeks after stopping NA therapy, out of 67 enrolled patients, 45 were randomly allocated to stop therapy. Of these patients, 17 patients required retreatment by week 72. The remaining 71% achieved virological relapse, and only 2% achieved HBsAg loss. This supports the notion that stopping NA will not confer long-term benefits. Most importantly, those who didn't achieve HBsAg loss were more susceptible to requiring retreatment and critically may lead to hepatic decompensation. This large prospective RCT, consisting mostly of Asian participants, strengthens the notion that stopping NA therapy, especially before HBsAg loss, is not beneficial but poses a risk of complications and retreatment, which is an unfavorable outcome. Contrary to another study, done predominantly in white people, shows that discontinuation of NA in HBeAg-negative patients will lead to a functional cure, i.e., HBsAg loss [18]. Out of 69 patients, only one developed HCC, as he had cirrhosis at baseline. 

Although the loss of HBsAg is considered a functional cure and a predictor of therapy cessation, another study in Taiwan explored the role of HBcrAg along with HBsAg in clinical relapse [20]. The study included 135 CHB patients who had already achieved viral remission and undetectable HBV DNA. During a follow-up of 2.1 years (25.9 months), 56% developed clinical relapse, and only eight patients developed HBsAg loss; 91% of patients developed virological relapse (HBV DNA >2000IU/ml). They generated a SCALE-B scoring system which includes surface antigen, core-related antigen, age, ALT, and tenofovir for HBV to predict relapse after NA cessation. All factors play an independent risk in clinical relapse, no matter the level of HBsAg or HBcrAg (Hepatitis B core Antigen) at the end of therapy. Manolakopoulos et al. further support and agree with the recommendations that patients without advanced-stage liver disease should discontinue NA therapy after long-term viral suppression [24].

Duration of Treatment

Duration of treatment, stopping treatment, and observing remission is crucial. Among Asian patients in a study, more disease remission was observed in 1.3 years 72 weeks) after stopping NA [16]. In the study based on the Spanish population, just after 49 weeks of stop trial, most patients achieved HBsAg clearance with minimal progression [24]. This might indicate that the Asian population needs long-term therapy to develop remission more than the white population. It is to be noted that NA is only stopped after long-term (>3 years) therapy. During this period, if HBV DNA remains less than 2000 and ALT remains normal, only then can the decision to stop NA be taken. Relapse is observed in most HBeAg-negative CHB patients who discontinue NA therapy, which strongly predicts retreatment. Retreatment, if necessary, should be initiated in 12-24 months, depending on the severity of virological or clinical relapse. The patient's epidemiology and treatment characteristics do not predict NA relapses, so additional research is required in this field.

Immunomodulatory Markers to Predict Disease Relapse

It is proposed that immunomodulatory markers can be used to assess and predict the disease course in CHB patients in the clinical setting [25]. IL-21 is an important cytokine that can mediate an immune response by affecting B and T cells in various stages of the disease. IL-21 can directly inhibit HBV replication and can indirectly do so by suppressing IL-10 [25]. IL-10 is the main predictor of early spontaneous HBeAg seroconversion [26]. During treatment, the IL-21 level was assessed. The virological relapse rate was 57.1% at 52 and 104 weeks after discontinuance of entecavir. The findings in the study showed that IL-21 has a direct relation with HBsAg levels in the viral remission group as it maintains immune control after the discontinuation of the drug. But the level of IL-21 decreased in the relapse group as the immune system could not suppress the viral activity. Kranidioti et al. conducted a prospective multicenter study to investigate other potential biomarkers in HBeAg-negative patients [22]. Four genes showed low expression upon studying peripheral blood mononuclear cells RNA on discontinuation and years after stopping NA. Genes (IFNg, CCL4, IL-8, and FASLG) can potentially predict off-treatment remission as the immune system is turned down on stopping treatment and shows sustained HBV suppression upon NA discontinuation.

Combination Therapy 

The use of IFN-ALPHA has been largely suppressed upon the introduction of NA due to its side effects and unfavorable mode of administration. It's used by only <5% of patients who either do not tolerate long-term NA or have immune-active CHB [5]. For immune-active CHB, there are still options for tenofovir and entecavir [11]. The most important factor considering therapy with either of the three is their lack of resistance to long-term use. A multinational clinical trial showed that the rate of HBsAg loss was 10.1% (at week 120, i.e., 72 weeks post-treatment) higher in patients receiving TDF plus PEG-IFN for 48 weeks rather than those receiving monotherapy with either of them or combination for 16 weeks followed by TDF alone for 32 weeks [19]. Although this study and many others investigate combination therapy, it is not favorable for clinicians as both classes of drugs need surveillance of side effects. PEG-IFN therapy is mostly chosen on patient preference. Patients who can tolerate its wide side effects and have only mild to moderate chronic hepatitis [27]. This strategy's limitation and lack of additional benefits are why it's not recommended in current guidelines [7].

Finally, there is a need to develop new drugs that target different phases of the HBV life cycle and cccDNA reservoir and focus on innate host immunity. Using finite NA has become a high HBsAg loss rate standard, but future drugs should aim at an even higher rate.

Limitations of this systematic review are that all included articles were published within the last five years as guidelines change rapidly. Studies on CHB patients with other comorbidities, acute flare, seeking treatment for other comorbidities, and baseline advanced liver disease were not analyzed. Studies that were not in the English language and were not in the database search, articles that were unpublished, not peer-reviewed, or grey literature.

## Conclusions

Treatment for chronic hepatitis B is evolving rapidly with new antiviral drugs and regimens but is still unsatisfactory. Our goal was to study the current treatment regimen and assess its role in the possibility of disease remission and relapse. The paradigm is shifting from infinite long-life NA therapy to finite NUC therapy. Although this therapy has succeeded in suppressing HBV DNA, there are few chances of HBsAg loss. Moreover, reliable factors are studied to predict outcomes in patients who stop NA therapy. These include IL-21, CCL-4, IFNg, IL-8, FASLG. However, as studies continue to predict off-treatment events and predictors, the best possible is very low HbsAg and undetectable HBV DNA. Close monitoring of HBV DNA and ALT post-treatment can predict retreatment options. Retreatment options then depend on the patient's strict predefined criteria baseline characteristics to avoid lifelong retreatment decisions.
